# Real-world handheld smartphone videos for nystagmus detection in acute vertigo: a feasibility study

**DOI:** 10.1007/s00415-026-14253-7

**Published:** 2026-11-12

**Authors:** Elvira Cortese, Benjamin Duvieusart, Haider Khan, Terence S. Leung, Nehzat Koohi, Diego Kaski

**Affiliations:** 1 Department of Clinical and Movement Neurosciences, SENSE Research Unit, Institute of Neurology, University College London, London, UK; 2 Escuela de Fonoaudiología, Facultad de Medicina, Universidad de Valparaíso, Valparaíso, Chile; 3 Comprehensive Stroke Service, National Hospital for Neurology and Neurosurgery, Queen Square, London, UK; 4 Advanced Research Computing Centre, University College London, London, UK; 5 Department of Medical Physics and Biomedical Engineering, University College London, London, UK; 6 Hull York Medical School, University of Hull and University of York, Hull and York, UK

**Keywords:** Smartphone nystagmography, Eye-tracking, Acute vertigo, Stroke, Emergency department

## Abstract

**Background:**

Accurate identification of nystagmus is fundamental to the assessment of acute vertigo but remains challenging in emergency department (ED) settings. Although smartphone-based approaches have been proposed as scalable solutions, their feasibility under real-world acquisition conditions remains uncertain.

**Objective:**

This study aimed to evaluate the feasibility of detecting nystagmus from handheld smartphone videos acquired in a real-world ED.

**Methods:**

A subset of 67 videos from a published handheld smartphone-based acute vertigo dataset was analysed. A salient feature-based eye-tracking algorithm tracked stable ocular and periocular (facial) landmarks across frames. Horizontal displacement signals were extracted, and nystagmus episodes were identified through signal analysis and video verification.

**Results:**

Consistent eye-movement signals were successfully extracted from handheld smartphone recordings acquired during the acute phase of vertigo despite real-world challenges, including variable illumination, camera motion, image instability, and patient-related factors. Nystagmus patterns observed on video were accurately reflected in the corresponding signal traces, including very low-amplitude nystagmus that was barely discernible to the naked eye.

**Conclusions:**

Our findings demonstrate that handheld image acquisition using standard, low-cost smartphones can capture clinically meaningful ocular motor information in real-world emergency care settings. This proof of principle lays the groundwork for the development of more ecologically valid scalable, telehealth-compatible tools for acute vertigo assessment. Further studies are needed to automate detection and validate performance in larger prospective cohorts.

## Introduction

Acute vertigo diagnosis in the emergency department (ED) presents a significant diagnostic challenge, partly because common vestibular disorders—such as vestibular migraine—can closely mimic serious life-threatening central causes such as posterior circulation stroke [1, 2]. Diagnosis relies mainly on skilled bedside assessment of subtle signs—including nystagmus, other abnormal eye movements, gait instability, and sudden hearing loss [3]. Although in the United Kingdom, the National Institute for Health and Care Excellence (NICE) recommends urgent specialist assessment for patients with focal neurological signs [4], implementation of these recommendations in emergency departments is often hindered by a range of factors, including limited specialist availability, training gaps, and time pressures, thus leaving frontline clinicians to make complex high-stakes decisions with substantial risk of both over-investigation and missed central pathology [1, 5, 6].

Nystagmus is a relevant clinical sign which, when present, can substantially help rapidly differentiate between central and peripheral conditions [7, 8]. The systematic and reliable identification of nystagmus through direct clinical observation remains a complex task, requiring specialised knowledge and a high level of clinical expertise to be performed accurately and consistently [9]. Alternatively, accurate recording of nystagmus is possible using video nystagmography or video-oculography tools; however these systems are also highly specialised, expensive, and impractical for widespread use in the emergency department settings [10–13].

There is a growing need for fast, accurate, affordable, and easy-to-use tools capable of detecting nystagmus and other subtle eye movements in real-world emergency department (ED) settings, thereby supporting frontline clinicians in the diagnosis of acute vestibular presentations [5, 6]. In this context, smartphone technology is particularly well suited due to its affordability, accessibility, ease of use, and widespread availability [10, 14–16]. These characteristics position smartphones as a promising platform for eye-movement analysis using classical computer vision and/or artificial intelligence (AI) and machine learning (ML) techniques, with the potential to improve the differentiation between central and peripheral vestibular disorders in the ED [10, 13, 17].

Over the past decade, interest in the feasibility of integrating smartphone-based technologies into healthcare has grown exponentially [18, 19], driving the development of applications for nystagmus detection and the diagnosis of acute vertigo and other vestibular disorders [20, 21]. Several smartphone-based prototypes have been developed to detect nystagmus and other eye movements [10–12, 17]; however, these systems have predominantly been trained and validated using highly controlled datasets, including recordings acquired with image-stabilisation devices (e.g. tripods), eye movements elicited through optokinetic stimulation, or synthetically generated data.

Although these approaches are methodologically valuable for proof-of-concept development, they do not adequately reflect the clinical and environmental conditions encountered during the assessment of patients with acute vertigo in the emergency department (ED), limiting their ecological validity and real-world applicability. This highlights an important translational barrier: the limited availability of real-world clinical data for developing and evaluating these technologies. Methods developed under controlled conditions may not generalise to routine clinical practice, where differences in patients' characteristics, recording conditions, and clinical environments can substantially affect performance. This gap between experimental and deployment settings is increasingly recognised as a major challenge to the clinical translation of digital health technologies [22, 23].

This is particularly relevant in the ED, where high patient volumes, clinical complexity, time constraints, and limited assessment space create a challenging and highly variable environment [5, 6, 24]. Smartphone-based nystagmography intended for ED use should therefore be developed and evaluated using data that reflect these conditions [5, 23, 25, 26].

As an initial step towards addressing this gap, we developed a real-world dataset of handheld smartphone videos from patients with acute vertigo assessed in the ED and made it publicly available through Zenodo [27]. However, whether nystagmus can be identified and tracked from such unconstrained, unfiltered real-world handheld smartphone videos remains unclear. This study therefore evaluates the feasibility of identifying nystagmus using this real-world ED dataset [27]. Establishing this feasibility not only would provide preliminary evidence of the utility of this particular real-world dataset, the Smart-vertigo dataset, but also would represent an important step towards the development of clinically applicable smartphone-based diagnostic support tools for acute vertigo.

## Methods

### Data

**Fig. 1:**
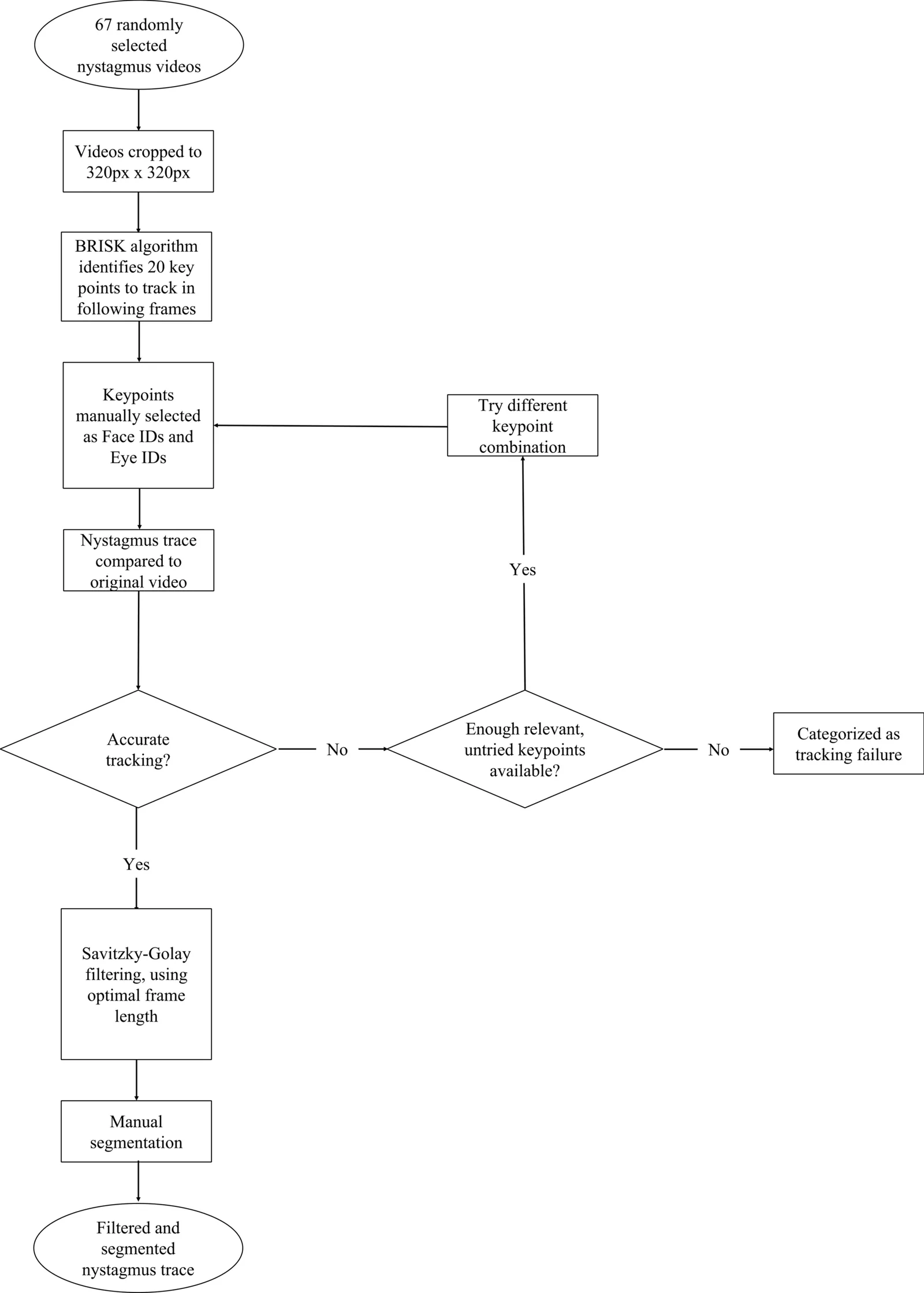
Flowchart showing the nystagmus tracking and segmentation process. *Px* pixels; *BRISK* binary robust invariant scalable keypoints. Savitzky–Golay filtering was applied using an optimal frame length, defined as the widest frame length that did not produce visible signal distortion. Accurate tracking involved comparison of the nystagmus trace with the input video looking for discrepancies in nystagmus beat direction, amplitude, and number of beats

A four-stage pipeline was implemented to extract digital nystagmus traces from handheld smartphone videos: data collection, salient feature tracking, signal filtering, and manual waveform segmentation. The overall workflow and its iterative processes are illustrated in Fig. 1.

The video data for this study are a subset of the SMART-VERTIGO database published by Cortese et al. [27].

### The smart-vertigo dataset

The dataset comprises 1,333 short, cropped eye-movement videos (3–5 s; > 400.000 RGB frames) acquired using a smartphone-based handheld approach, from 100 patients with acute vertigo assessed in a UK tertiary-hospital ED during acute presentation of symptoms (57 female, 43 male; aged 18–97 years; 51% white, 18% Asian, 17% Black, and 14% mixed/other). Videos were recorded at 30 and 60 frames per second (fps) and were pseudonymised by cropping them to the region of interest (ROI), thereby minimising the risk of disclosing sensitive information.

All videos were recorded under real ED conditions, reflecting routine clinical variability in lighting conditions, patient characteristics and cooperation, and recording stability. They were recorded using the same mid-range Android smartphone (Galaxy A55), without stabilisers or additional hardware. Recordings and acute neurological bedside assessments were performed by a single experienced vestibular specialist (EC), who documented the initial diagnostic impression and video-observable oculomotor findings, including the presence and direction of nystagmus. These preliminary assessments were subsequently revised by two senior vestibular specialists (DK and NK), who established final clinical diagnoses according to Bárány Society diagnostic criteria.

### Data subset for assessing the feasibility of nystagmus identification

For the present study, 67 gaze-test videos from patients in which nystagmus had been clinically identified were randomly selected from the subset of the Smart-vertigo dataset containing pathological nystagmus [27].

To assess the feasibility of analysing real-world ED smartphone recordings, we applied a salient feature-based eye-tracking method as a deliberately simple and interpretable analytical approach. Rather than maximising detection performance, this method was designed to assess the intrinsic quality of the video signal by evaluating whether clinically meaningful nystagmus information could be reliably extracted from unconstrained, unfiltered handheld smartphone recordings.

For video-based analysis, recordings were required to be technically trackable and to show sufficiently appreciable nystagmus, defined as more than two visible and consecutive beats. Videos in which tracking was not possible or in which two or fewer nystagmus beats were appreciable were excluded from further analysis.

Eye-tracking success was defined as the presence of sufficient number of suitable keypoints to enable tracking by the salient feature algorithm. Nystagmus was considered correctly identified when it was visually appreciable in the source video, the tracking output demonstrated nystagmus in the corresponding horizontal direction, and the eye displacement represented by the tracking trace was visually consistent with that observed in the source video. Tracking performance was summarised descriptively, and no formal statistical comparisons were performed between frame-rate or nystagmus type groups owing to the small and unequal number of recordings in each group, and because these comparisons were beyond the scope of this feasibility study. Nystagmus-negative controls were not included at this stage; therefore, the ability of the algorithm to identify the absence of nystagmus was not assessed.

### Salient feature tracking

Eye tracking was performed using the Open-Source Computer Vision library (OpenCV) via Python 3. Features were identified using Binary Robust Invariant Scalable Keypoints (BRISK) algorithm. This algorithm firstly identities keypoints using the FAST (Features from Accelerated Segment Test). This uses changes in pixel intensity, to identify possible edges and corners and their relative “strength”. This is repeated at different scales to estimate the “size” of an edge/corner. 20 points with the highest scores continue to be tracked in subsequent frames. BRISK then compares intensity of pairs of pixels around each keypoint, where if sample 1 within a pair is brighter than sample 2 it is assigned a value 1, otherwise assigned 0. This is repeated to build a 512-bit binary descriptor for each keypoint. For example, a keypoint may be assigned a descriptor of [10110011…] and in the next frame a keypoint is assigned as [10111011…]. The Hamming distance in this example, that is the number of dissimilar bits, is 1. Therefore, it is assumed that this keypoint is the same anatomical feature, allowing tracking of a feature across frames despite changes in scale and rotation [28].

**Fig. 2:**
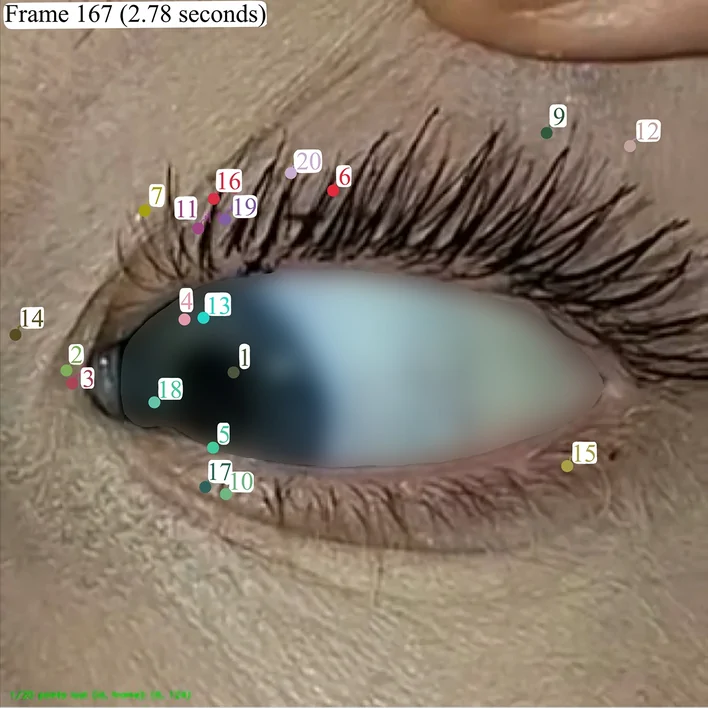
Salient feature tracking applied to nystagmus video. Numbered keypoints were available for manual selection, as either Face ID or Eye ID. In this example, points 1, 4, 13, and 18 are suitable candidates for Eye IDs. Points 2, 3, 12, and 14 are suitable candidates for Face IDs. Eye blurred for anonymity. Unblurred video available via SMART-VERTIGO database [27]

Since keypoints were detected based on pixel-level characteristics rather than semantic information, manual supervision was required. After keypoints detection and tracking, the 20 keypoints with the highest response scores were presented for manual selection and classification as either “Face IDs” or “Eye IDs”, as illustrated in Fig. 2. Each keypoint was expressed as two-dimensional image coordinates (x, y). When multiple Eye IDs or Face IDs were selected, their x and y coordinates were averaged separately to obtain the centroid coordinates for the eye (Mean Eye ID x, Mean Eye ID y) and face (Mean Face ID x, Mean Face ID y). Face IDs were stationary landmarks such as the lateral canthus, eyebrows, cutaneous naevi, and eye lashes, and were used as a spatial reference object for the camera motion. Eye IDs were keypoints on moving ocular landmarks such as scleral vasculature, limbus, iris crypts, and pupillary margin. For example, in Fig. 2, keypoints 1, 4, 13, 18 are candidate Eye IDs, whereas points 2, 3, 12, and 14 are candidate Face IDs. If the eye centroid was located at (15, 100) and the face centroid at (10, 80), the resulting net relative eye position would be (5, 20). The calculations reduced the contribution of head or camera movement to the estimated eye displacement as shown in Eq. 1.

$$Mean ~Eye~ ID ~x = \frac{1}{N} \sum_{i=1}^{N}Eye ~ ID~ {x}_{i},$$

$$Mean ~Face~ ID~ x = \frac{1}{N} \sum_{i=1}^{N}Face~ ID~ {x}_{i},$$

Netx=MeanEyeIDx-MeanFaceIDx.

**Fig. 3:**
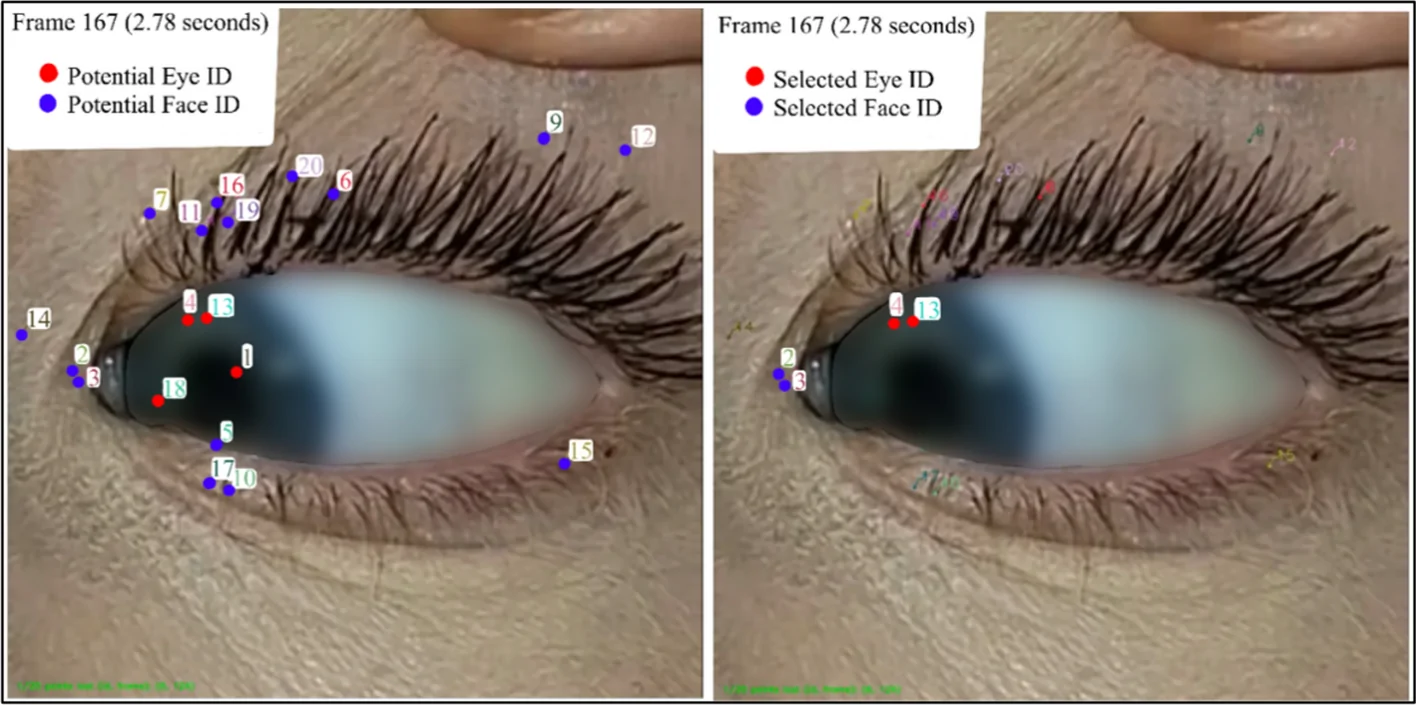
Keypoints selection process. The left panel shows the candidate keypoints for Eye IDs (red), and Face IDs (blue). The right panel highlights the selected keypoints for tracking. In this example, keypoints 2 and 3 were selected as Face IDs as they were a stable landmark compared to keypoints on mobile landmarks like the eyelashes (7,11,16,19…)

**Fig. 4:**
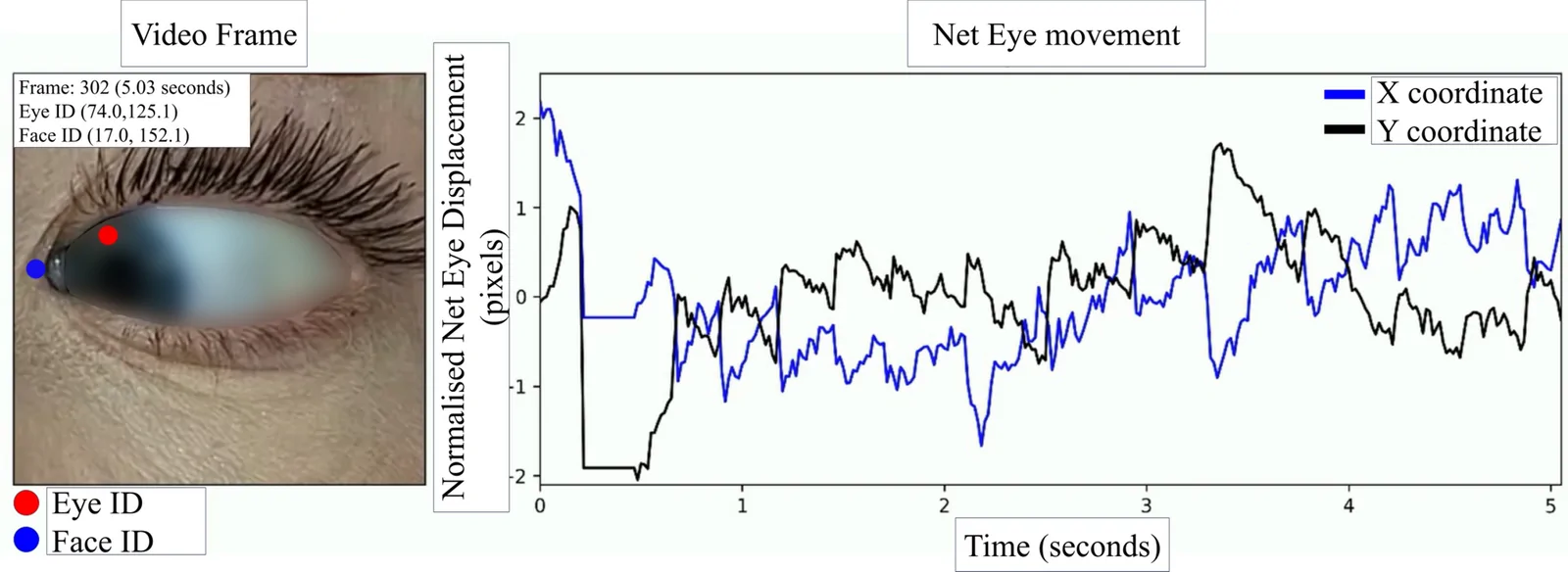
Output video post salient feature tracking. The nystagmus trace on the right was qualitatively compared to the video on the left to ensure accurate tracking

Equation 1 Net eye-movement equations—Eye ID, keypoint with image coordinates (x, y) manually selected to track a moving ocular landmark. Eye ID x, horizontal component of the Eye ID coordinate. Mean Eye ID x, mean average of several Eye ID x. Face ID, keypoint with image coordinates (x, y) manually selected on a stationary landmark as an analogue for camera movement. Face ID x, the horizontal component of the Face ID coordinate. Mean Face ID x, mean average of several Face ID x. Net x, true horizontal eye displacement with the camera movement removed. N is the number of selected Eye IDs or Face IDs Figs. 3 and 4.

**Fig. 5:**
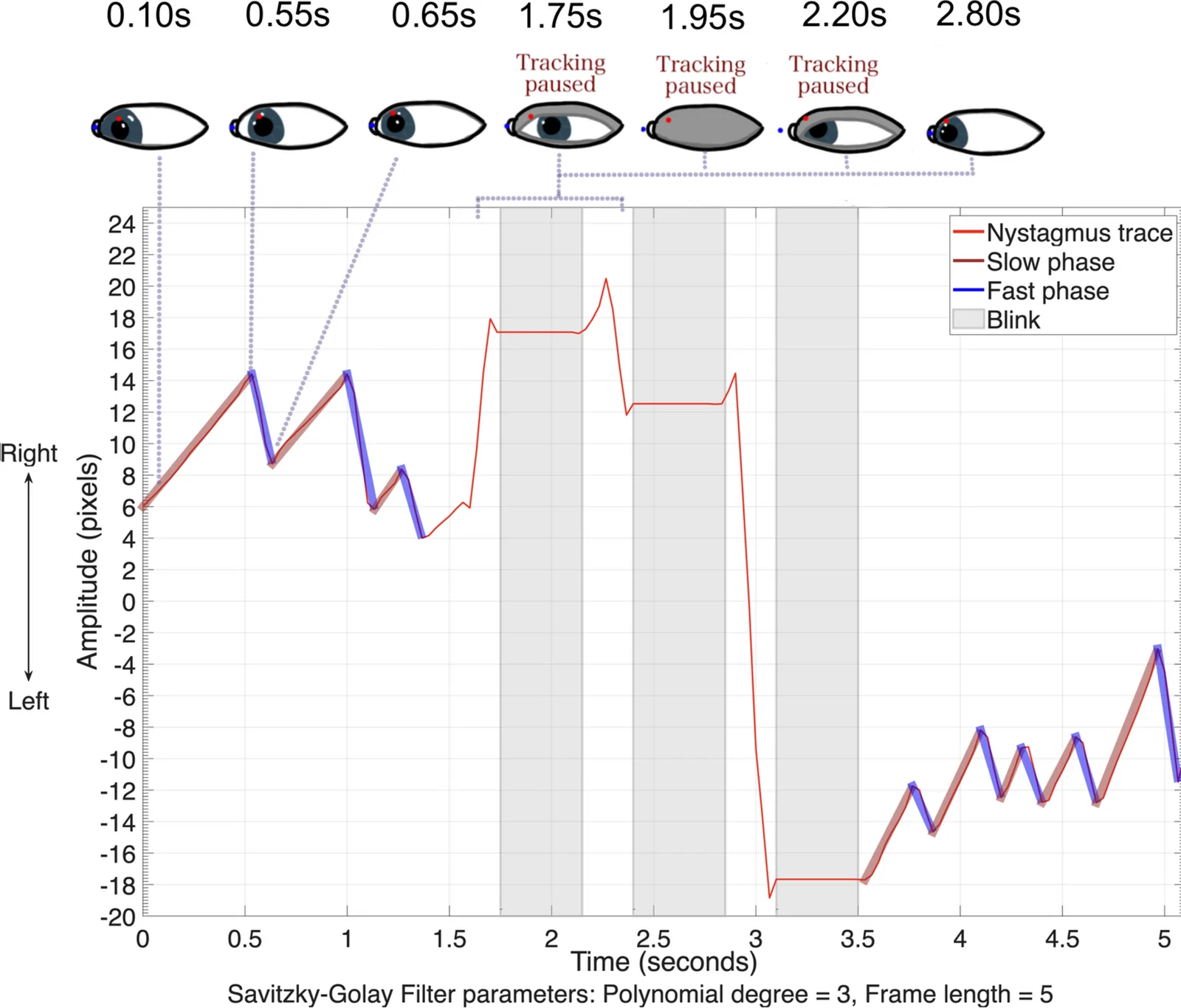
Horizontal component of the filtered nystagmus and corresponding eye-tracking signal. The figure illustrates the relation between horizontal eye movements and the corresponding nystagmus trace over time (red line), highlighting the tracking-pause mechanism implemented during blinks. The x-axis represents elapsed recording time (s), and the y-axis represents horizontal eye displacement in pixels, with increasing and decreasing values indicating rightward and leftward movement, respectively. The thin red line depicts measured eye displacements over time. Blue and brown highlights segments indicate the fast-phase and slow phases of nystagmus beats, respectively. Grey-shaded regions indicate blinking intervals during which eye tracking was temporarily paused. Eye illustrations on top depict the corresponding eye positions and tracking status at different time points, where Blue and red dots represent the FaceID and EyeID points, respectively. During blinks, tracking is temporarily interrupted, potentially obscuring nystagmus beats and resulting in keypoint drifts. Following each blink, the keypoints return to their original anatomical landmarks, allowing tracking to resume. The signal was processed using a Savitzky–Golay filter with a polynomial degree of 3 and a frame length of 5

To address a limitation of the BRISK algorithm, which requires landmarks to remain visible throughout the video, an automatic blink detection method was implemented. This approach aimed to mitigate tracking failures caused by keypoints occlusion or movement outside the camera’s fields of view, which could result in an insufficient number of suitable keypoints to reliable tracking. This method incorporated a 0.5 s pause in tracking, allowing for a complete blink to occur for the landmark to become visible to the camera again. In the resulting nystagmus trace, successfully detected blinks were represented by a flat segment in the eye-position trace (Fig. 5). Consecutive blinks could result in longer tracking interruptions and increase the likelihood of keypoint displacement or loss [28].

### Visual assessment of tracking performance

The resulting nystagmus traces were visually inspected by HK, an engineering student who has experience in computer vision techniques and received brief training in nystagmus recognition from EC, a vestibular specialist experienced in interpreting nystagmus from video nystagmography (VNG) and bedside examination. Each nystagmus trace was compared with the corresponding source video to assess whether the extracted eye movements were consistent with the observed nystagmus (Fig. 4). If tracking was inadequate, keypoint combinations were iterated upon until a satisfactory tracking of the eye was performed. If no combination of keypoints was able to reliably represent the nystagmus trace, the video was categorised as a tracking failure and excluded from analysis.

### Nystagmus segmentation

The filtered traces were manually segmented by HC and later revised by EC. Each segmented trace was reviewed against the corresponding source video to confirm that the identified segments represented nystagmus rather than other eye movements or artefacts, such as square-wave jerks or blinks. This process integrated computer vision and clinical expertise to improve the clinical plausibility of the final nystagmus segmentation. Discrepancies between computational results and clinical criteria were jointly reviewed and discussed, leading to refinements in the classification through a consensus-based approach. Clinical interpretation was informed by the vestibular specialist.

Fast-phase direction was interpreted according to pixel-based coordinates, which are inverted relative to conventional VNG/VOG displays.

The reviewers manually annotated the onset and offset of the slow and fast phases. As aliasing and the Savitzky–Golay filtering introduced uncertainty in identifying the boundaries between these phases, a standardised annotation protocol was adopted, to ensure consistent phase identification and segmentation. This protocol avoided data interpolation and prioritised the preservation of slow-phase morphology, given its greater diagnostic relevance [29].

In this study, only the horizontal component of eye movement was analysed, as it exhibited a higher signal-to-noise ratio (SNR) than the vertical component, enabling more reliable tracking. The raw nystagmus traces were filtered with a Savitzky–Golay filter, which applies least square fitting of a third-degree polynomial to consecutive subsets of data. The frame length was selected iteratively for each trace, as outlined in Fig. 1, starting from a minimum frame length of 3, and progressively increasing it until visible signal distortion was observed. The preceding, and therefore widest non-distorting, frame length was selected for filtering. No distortion was observed at minimum frame length of 3 in any of the traces analysed; had this occurred, the raw, unfiltered trace would have been used for segmentation. The Savitzky–Golay filter was utilised for its simplicity and its ability to reduce high-frequency noise while preserving rapid signal transitions [30]. The temporal relationship between horizontal eye movements and the corresponding nystagmus trace is illustrated in Fig. 5.

## Results

Of the original 67 randomly selected videos, 13 were recorded at 30 fps and 54 at 60 fps. Initial tracking was possible in 63/67 videos (94%); 13/13 at 30 fps (100%) and 50/54 at 60 fps (92.6%), respectively, which contained sufficient visual information for subsequent analysis despite non-ideal real-world acquisition conditions.

**Fig. 6:**
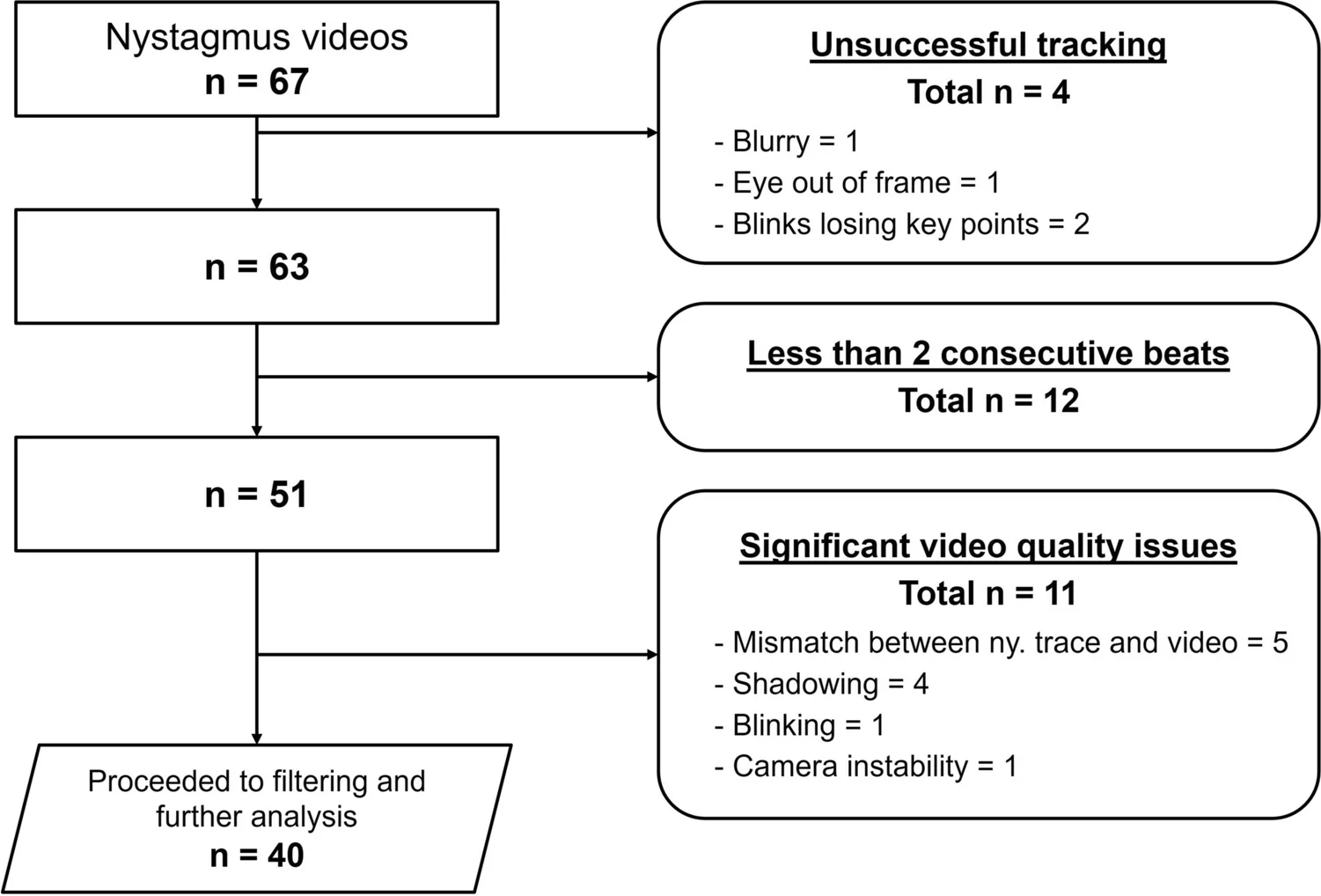
Flow diagram of video recording selection. Unsuccessful tracking means insufficient or inappropriate keypoints for salient feature tracking. *ny* nystagmus

While videos were randomly selected from recordings of patients with clinically confirmed nystagmus, some did not meet the minimum requirements for reliable nystagmus tracking, as having a minimum of 2 consecutive beat in the recording. Additionally, videos having significant blinking and/or shadowing were excluded from further analysis. 40 videos (72.7%) complied with requirements for nystagmus tracking (12/13 at 30 fps [92.3%] and 28/54 at 60 fps [51.9%]) and were included in the final analysis (Fig. 6).

Figure 6 illustrates the flow of video recordings selection through the eye-tracking assessment process, detailing the reasons for exclusion at each stage and the number of recordings retained for further analysis.

**Fig. 7:**
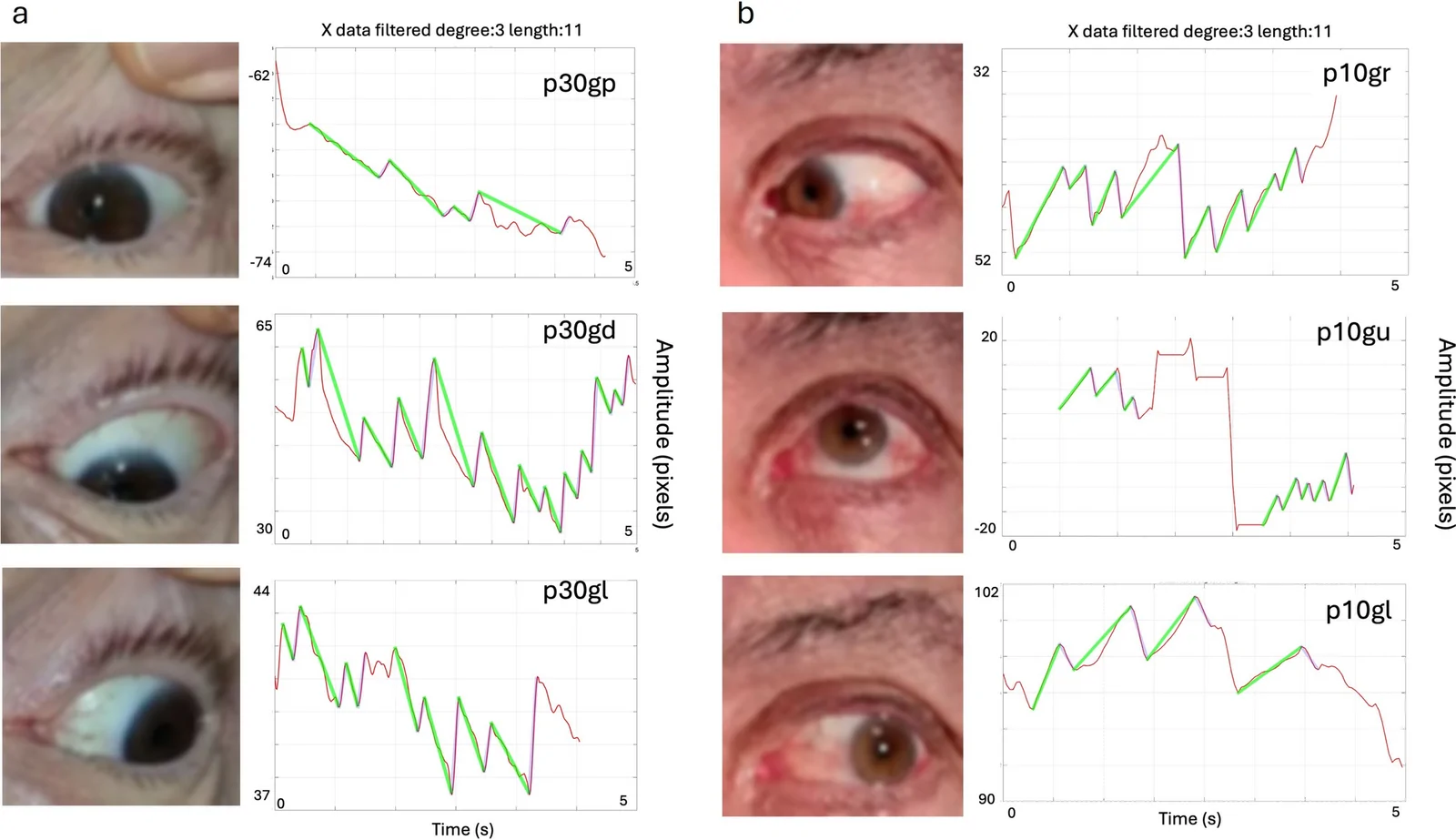
Third-degree spontaneous nystagmus tracings from ward recordings. **A** Case p30av: low-amplitude left-beating nystagmus. **B** Case p10av: low-amplitude right-beating nystagmus recorded under sub optimal ward lighting. Both are consistent with third-degree nystagmus according to Alexander’s law: nystagmus is present in primary gaze and in both gaze directions, increasing when gazing towards the fast phase and decreasing when gazing away from it [7]. Tracings are inverted relative to conventional VNG/VOG displays: upward fast-phase deflections indicate left-beating and downward deflections right-beating nystagmus. Videos were blurred to protect patient identity. Video codes: p30 and p10 (Zenodo). Abbreviations: gp, primary gaze; gd, downgaze; gu, upgaze; gl, left gaze; gr, right gaze

**Fig. 8:**
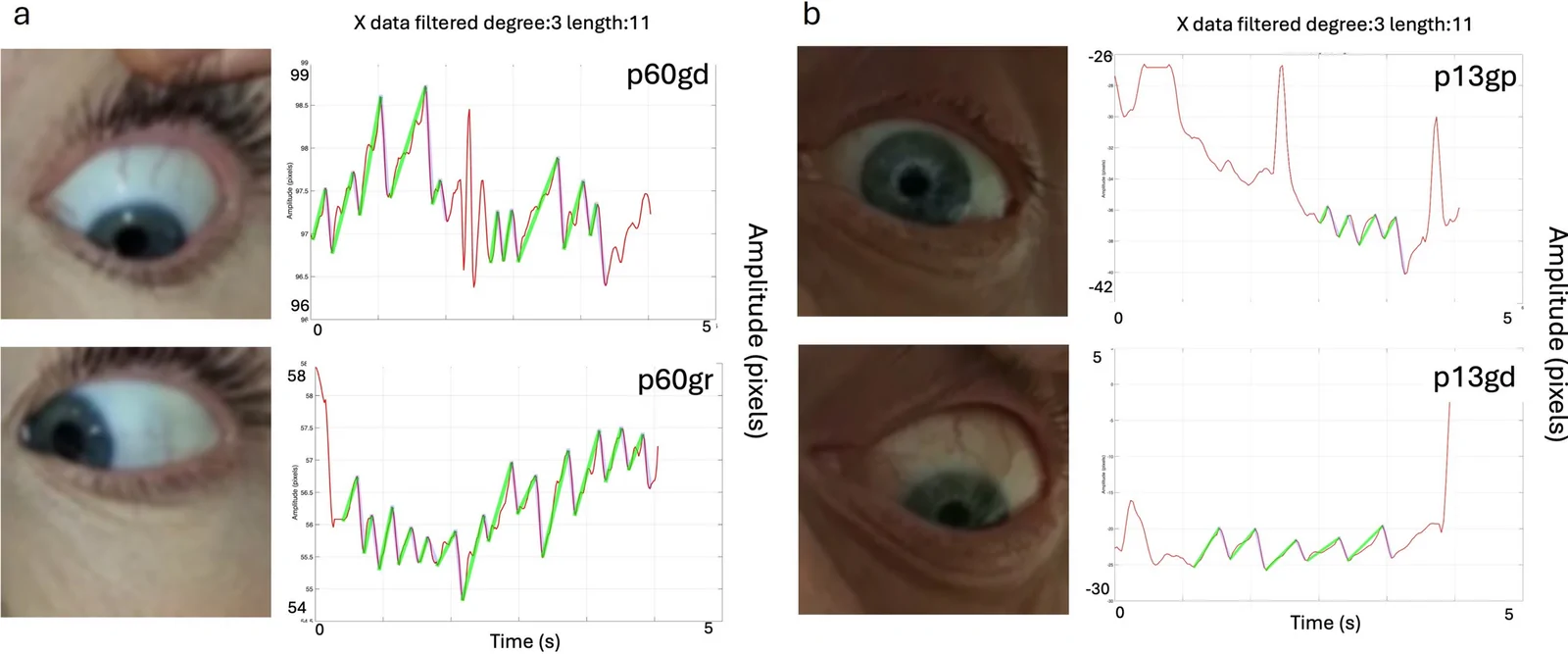
Low-amplitude spontaneous nystagmus tracings from ward recordings. **A** Case p60av: right-beating unidirectional nystagmus, visible in gaze down and in gaze right. **B** Case p13av: right-beating nystagmus recorded under poor lighting conditions and shadowing, with unidirectional nystagmus present in primary gaze and downgaze. As these are pixel-based tracings, fast-phase direction is inverted relative to conventional VNG/VOG displays: upward deflections indicate left-beating nystagmus and downward deflections of fast phases indicate right-beating nystagmus. Videos were blurred to protect patient identity. Video codes: p60 and p13 (Zenodo). *gp* primary gaze; *gd* downgaze; *gr* right gaze

Nystagmus was also successfully identified in recordings with very low-amplitude eye movements, under suboptimal lighting conditions with shadowing, in the presence of noticeable scene or camera motion, and in participants wearing eye makeup. Representative examples illustrating these challenging scenarios are shown in Figs. 7 and 8. Full-length videos are available in the Zenodo repository [27].

### Limiting factors

Blinking emerged as the primary factor limiting performance, interrupting signal acquisition and interfering with nystagmus identification within the current algorithmic pipeline. Nevertheless, the algorithm rapidly resumed tracking after blinks, successfully reacquiring eye landmarks even when their positions had shifted substantially. In several instances, tracking resumed after the blink despite temporary displacement of the tracked keypoints, allowing a usable nystagmus trace to be obtained even when multiple blinks occurred within the video. Other factors such as reflections, unstable footage —as video containing unintended handheld camera motion (e.g. shake or jitter)— and suboptimal pupil visibility were present but less consistently associated with failure.

## Discussion

Taken together, these findings demonstrate that real-world handheld smartphone eye-video recordings acquired in the emergency department (ED) [27] constitutes a viable and clinically meaningful data source for automated nystagmus detection. Despite the inherent variability and artefacts associated with routine clinical practice, most recordings were usable with nystagmus successfully detected in a substantial proportion of cases.

Representative recordings further demonstrated that accurate eye tracking is achievable under conditions that are typically challenging for unaided visual assessment. Robust detection was maintained despite low-amplitude eye movements, suboptimal illumination, shadowing, and substantial camera or scene motion. These findings suggest that factors often considered major barriers for nystagmus tracking methods and for the use of real-world videos—such as variable lighting, motion artefacts, and reduced signal amplitude [31]—are not the principal determinants of detection failure.

Instead, unsuccessful detections were more consistently associated with transient loss of signal, coming from transient ocular occlusions, particularly blinking, which interrupt the continuity of salient visual features [33]. This limitation appears to reflect the sensitivity of the current feature-based tracking approach rather than deficiencies in the quality of handheld smartphone recordings themselves. Future improvements are therefore likely to depend on tracking strategies that maintain stable eye-region representations during partial occlusion, such as segmentation-based approaches or temporally robust tracking algorithms [33].

Recent advances in mobile health technologies have demonstrated the feasibility of smartphone-based eye-movement assessment, highlighting the potential to improve the diagnosis of neuro-otological disorders [10–13, 32]. However, progress remains constrained by the lack of publicly available datasets containing handheld smartphone recordings of eye movements from patients presenting with acute vertigo under real-world ED conditions. Practical and ethical challenges surrounding data collection during acute clinical presentations have limited the availability of such resources, thereby restricting the development and validation of ML models capable of recognising clinically relevant oculomotor patterns [23, 26, 34–36].

The use of real-world handheld smartphone recordings is particularly relevant to acute vertigo assessment, where distinguishing potentially life-threatening central causes, such as stroke, from more common peripheral vestibular disorders remains challenging because of overlapping clinical presentations and limited familiarity with nystagmus among non-specialist clinicians. Training and validating eye-tracking methods using real-world ED smartphone recordings offer a more realistic pathway towards clinically deployable diagnostic systems than development based on controlled laboratory datasets. By capturing clinically relevant eye movements under authentic operating conditions, real-world datasets are more likely to produce models that generalise effectively to routine practice [22, 37, 38].

Furthermore, smartphone-based data acquisition [27] provides a scalable, accessible, and low-cost platform that avoids reliance on specialised hardware, creating opportunities to expand access to neuro-otological assessment, particularly in resource-limited settings [20]. In this context, AI-assisted smartphone tools could contribute to reducing healthcare inequalities by supporting earlier, more accurate triage of patients with acute vertigo, including improved identification of high-risk central causes such as stroke [15, 16, 39].

As highlighted earlier in Discussion, clinically representative real-world data are essential for translating technological advances developed under controlled experimental conditions into routine clinical practice. Addressing this challenge is a key contribution of the present study. By developing an open-source dataset from patients assessed under routine ED conditions and subsequently demonstrating that these data can be used to identify and track nystagmus-related eye movements, our work provides a framework for developing and evaluating computational methods in conditions that more closely reflect their intended clinical environment. This may help reduce the domain shift that occurs when methods developed using highly controlled data are transferred to real-world settings [22, 23, 40].

Our findings provide preliminary evidence that the dataset contains sufficiently informative eye-movement data for computational analysis and nystagmus identification, supporting its intended purpose [27]. They also support affordable, handheld smartphones as a practical platform for collecting clinically representative eye-movement data. Their affordability and widespread availability may, in turn, facilitate broader access to future diagnostic decision-support tools[14, 19, 41].

More broadly, our findings highlight the value of open-access, real-world datasets for developing and evaluating computer vision and machine learning methods for automated eye-movement analysis [23, 25, 26, 34, 36]. Demonstrating the feasibility of nystagmus identification under real-world ED conditions therefore represents an important step towards clinically relevant, scalable, and accessible smartphone-based diagnostic support systems [22, 37, 38]. Future studies should validate the utility of this real-world video material for nystagmus identification and eye-movement analysis against established reference methods such as VNG/VOG, using this or alternative computational approaches, including binary semantic segmentation of video frames.

Finally, the limitations observed within this real-world handheld smartphone dataset primarily reflect the practical constraints of the ED environment and the recording modality itself—the same conditions under which future smartphone-based eye-tracking tools will ultimately operate. Consequently, these characteristics should be regarded as important aspects of ecological validity rather than simply technical shortcomings. As signal processing, computer vision, and machine learning methods continue to advance, many of the current limitations are likely to be mitigated, further improving robustness and supporting the integration of smartphone-based eye-tracking tools into routine emergency care.

Future work should extend the current approach to the quantitative assessment of vertical nystagmus and torsional component as an important next step towards more comprehensive eye-movement characterisation. Further development should also focus on automating the two stages of the current tracking pipeline that require manual input, thereby reducing operator dependence and improving reproducibility and scalability. These developments would provide a foundation for subsequent incorporation of more complex quantitative analyses, including identification and characterisation of pursuit and saccadic eye movements and the calculation of nystagmus dynamics such as slow-phase velocity variability and temporal irregularity, which may provide more discriminative markers for differentiating central from peripheral vestibular disorders.

### Limitations

The main limitation is the absence of a ground-truth reference for eye movements. Although the proposed method tracks nystagmus, no baseline was available against which tracking accuracy could be quantitatively evaluated. Consequently, successful detection was assessed qualitatively rather than through objective performance metrics [42]. This limitation reflects the design of the study that generated the master dataset, whose primary objective was to replicate the ED assessment of patients with acute vertigo using bedside, and deliberately excluding objective eye-movement assessment techniques [27]. As the present feasibility study was designed after the parent dataset has been developed, no parallel VNG or VOG data were available for gold-standard comparison, which was also beyond the scope of this exploratory feasibility study. Given the small and unevenly distributed subgroups, tracking performance was not evaluated separately by nystagmus type; future studies with larger datasets should assess performance across spontaneous, gaze-evoked, and positional nystagmus.

Another important limitation of this study is that only 40 of the 67 initially selected recordings (60%) met the criteria for reliable nystagmus analysis, despite successful initial eye tracking in 94% of the videos. Exclusions were mainly due to insufficient consecutive nystagmus beats, blinking, or shadowing. This highlights the challenges of obtaining handheld smartphone recordings of sufficient quality for quantitative nystagmus analysis under real-world emergency department conditions, which may potentially limit the clinical applicability of the proposed approach. Standardised recording protocols and improved acquisition conditions may help overcome these limitations.

Moreover, although smartphone camera technology has advanced considerably in recent years, important technical limitations remain in the accurate capture of high-velocity oculomotor events, particularly rapid saccadic eye movements (typically 300–700 deg/s) [43]. The limited temporal resolution of standard smartphone video acquisition may reduce the accuracy of estimating dynamic ocular motor parameters, including saccade onset, peak velocity, and trajectory [44, 45].

Nevertheless, continued improvements in mobile imaging, sensor resolution, and computational photography are expected to alleviate these limitations, further enhancing the clinical feasibility of smartphone-based diagnostic systems. As these technologies mature, they have the potential to increase access to neuro-otological assessment and facilitate integration into routine emergency care pathways [10, 15, 46].

Finally, although the dataset provides a robust foundation for the development of classical computer vision eye-tracking methods, further validation is required to establish generalisability and clinical utility. Future work should investigate computer vision and machine learning approaches that reduce reliance on manual input, including binary semantic segmentation of eye frames and automated landmark detection for eye-tracking. These approaches could facilitate fully automated eye-movement extraction and nystagmus detection, while maximising the dataset’s value for developing and benchmarking robust, scalable smartphone-based tools for the assessment of acute vertigo in emergency care.

## Conclusion

This study demonstrated the feasibility of identifying nystagmus from annotated handheld smartphone-based videos acquired under routine emergency department conditions [27]. The dataset incorporates several features inherent to real-world clinical assessment, including demographic variability, image instability, variations in illumination, variable camera positioning, and video resolution. Although these factors introduce technical challenges, they also reflect the conditions under which smartphone-based eye-movement assessment would be applied in clinical practice. This real-world dataset therefore provides a clinically relevant basis for further investigation of automated nystagmus assessment from smartphone video.

## Data Availability

The pseudo de-identified videos analysed in this study are publicly available in the Zenodo repository at https://doi.org/10.5281/zenodo.18758864. The code used in this study can be made available upon request.
